# Effects of early estradiol valerate administration on bone turnover markers in surgically induced menopausal women

**DOI:** 10.1186/s12905-021-01508-w

**Published:** 2021-10-13

**Authors:** Jarika Vatrasresth, Ammarin Suwan, Krasean Panyakhamlerd

**Affiliations:** 1 Reproductive Medicine Division, Departments of Obstetrics and Gynecology, Faculty of Medicine, King Chulalongkorn Memorial Hospital, Chulalongkorn University, 1873 Rama IV Rd. Pathum Wan, Pathum Wan District, Bangkok, 10330 Thailand; 2 Gender, Sexual and Climacteric Medicine Division, Departments of Obstetrics and Gynecology, Faculty of Medicine, Chulalongkorn University, Bangkok, Thailand; 3 Center of Excellence in Transgender Health, Chulalongkorn University, Bangkok, Thailand

**Keywords:** Surgical menopause, Bone turnover markers, Serum CTX, Serum P1NP, Estradiol valerate

## Abstract

**Background:**

Compared with a natural process, surgically induced menopausal women have a higher bone loss rate. This study aims to evaluate early treatment with estradiol valerate on bone turnover markers after surgically induced menopause.

**Methods:**

This prospective study included 41 pre and perimenopausal women who underwent hysterectomy with oophorectomy for benign gynecologic conditions. Two weeks after the operation, all participants were assessed for menopausal hormone therapy (MHT) indications. Estrogen therapy was prescribed for those who had indications and accepted treatment (hormone treatment group). The others who had no MHT indication were allocated to the no-treatment group. Serum CTX and P1NP levels at preoperative and 12 weeks postoperative were measured and set as the primary outcome. Within the same group, serum CTX and P1NP before and after surgical menopause were analyzed using Wilcoxon signed-rank test. ANCOVA was used to compare serum CTX and P1NP at 12 weeks after surgical menopause between the two groups. Spearman's rank correlation coefficient analysis analyzed the correlation between age and baseline bone turnover markers. A *p*-value of < 0.05 was considered statistically significant.

**Results:**

At 12 weeks after surgery, there were no significant differences in serum CTX and P1NP levels in the hormone treatment group compared to baseline. In contrast, serum CTX and P1NP levels were significantly elevated among women who did not receive hormone treatment (*p*-value < 0.001 and 0.002, respectively). Serum CTX and P1NP at 12 weeks were significantly different between the two groups (*p*-value < 0.001 and 0.004, respectively).

**Conclusion:**

Early estrogen administration with oral estradiol valerate could significantly suppress the high bone remodeling in surgically induced menopausal women.

*Trial registration* Thai Clinical Trial Registry identification number TCTR20190808004, retrospective registered since 2019-08-08. http://www.thaiclinicaltrials.org/show/TCTR20190808004.

## Background

Osteoporosis is one of the most critical health risks for postmenopausal women. Osteoporotic fractures, especially hip fractures, are associated with high morbidity and mortality rate [1]. The main determining factors of postmenopausal osteoporosis are peak bone mass status and rate of continuing bone loss [2, 3]. Estrogen deprivation during menopause is the primary cause of accelerating bone loss. Uncoupling the bone formation and resorption at this stage results in depletion of estrogen-mediated inhibition of the bone resorption [4].

*Natural menopause* is an aging process that occurs at 49.5 years in Thai women and around 52 years in the Western population [5]. It is defined as the permanent cessation of a menstrual period for more than 12 months and occurs when the ovarian follicles are depleted. Approximately 2–3 years before the final menstrual period, estrogen gradually declines concurrently with follicle-stimulating hormone (FSH) elevation.

In contrast, surgical removal of both ovaries before the onset of natural menopause results in a sudden loss of ovarian hormone production, including estrogens, progesterone, and testosterone [6, 7]. Possible evidence suggested that surgical menopause was associated with long-term adverse health consequences such as low bone mass, cardiovascular disease, cognitive impairment, and an increase in the overall mortality rate [8, 9]. Still, bilateral oophorectomy was regularly performed (at least in some countries) at the time of hysterectomy in peri or premenopausal women aged around 50 years. Currently, the concept of oophorectomy for ovarian cancer prevention in normal gross finding ovaries has been debated [10]. Our team concern on this controversial issue, especially acute bone loss after possible unnecessary oophorectomy. We aim to evaluate a dynamic bone status (bone remodeling) in surgical menopause and the protective effects of estrogen therapy for this condition.

*Bone remodeling* is a dynamic process originating from bone resorption (osteoclastic activity) and following bone formation (osteoblastic activity). During the remodeling process, several bone turnover markers are released. Bone turnover markers are classified into two main categories, bone resorptive and formative bone markers. The most commonly used bone resorption markers in clinical practices and researches are *cross-linked C*-*terminal telopeptide* of *type* I *collagen* (CTX) and N-terminal (NTX). In contrast, bone-specific alkaline phosphatase (BAP), osteocalcin, procollagen type 1 N-terminal propeptide (P1NP), and procollagen type 1 C-terminal propeptide (P1CP) can be used as bone formation markers [11]. Due to analytic variability and utility limitations of these bone turnover markers, the International Osteoporosis Foundation (IOF) and the International Federation of Clinical Chemistry and Laboratory Medicine (IFCC) recommend using only serum P1NP and CTX as bone formative and bone resorption markers in clinical practices, respectively [12].

The accelerated bone remodeling process in natural menopausal women resulted in elevated serum bone formative and resorptive markers levels and decreased bone mineral density (BMD) [13]. On the other hand, abrupt and significant declination of estrogen levels in surgical menopause was more pronounced. Loss of bone detected by BMD measurement was more affected than in the natural process [14]. Compared with natural menopausal women, surgically induced menopausal women had a relatively greater rate of bone remodeling and rate of bone loss [15].

Menopausal hormone therapy (MHT) was approved for menopausal women with at least one out of four indications, including relief of bothersome vasomotor symptoms, prevention of bone loss in women at high risk for fractures, prematurely hypoestrogenism, and genitourinary syndrome of menopause [16]. MHT regimens are classified into two main types, estrogen-alone, and estrogen-progestogen therapy. The estrogen-alone regimen is preferred in hysterectomized menopausal women. In non-hysterectomized menopausal women, the estrogen-progestogen regimen is indicated. The primary purpose of progestogen in the MHT regimen is for endometrial protection [16] because the chance of endometrial hyperplasia and endometrial cancer is significantly raised in non-hysterectomized women using the estrogen-alone regimen [17, 18]. However, current evidence demonstrated that women using estrogen-progestogen regimens had a higher risk of breast cancer and venous thrombosis than estrogen-alone regimens [19, 20]. For these reasons, estrogen-alone is the MHT regimen of choice for hysterectomized menopausal women.

The primary purpose of this study was to evaluate the effects of early estrogen-alone therapy with oral estradiol valerate on the levels of bone turnover markers (CTX and P1NP) after surgical menopause procedures. The reasons for choosing this form of estrogen in our research were availability of this medication in several countries, inexpensive cost, and estradiol valerate could be metabolized to 17β-estradiol by gastrointestinal tract enzymes in a comparable dose efficacy. Consideration for 2 mg of estradiol valerate is based principally on the recommendation from literature as moderate to high estradiol dose [21]. However, there are global variations in doses perceived as low, medium, and high [22].

## Methods

### Design

The study was designed as a single-center, prospective trial at the King Chulalongkorn Memorial Hospital Thailand between June 2019 and April 2020. This study was approved by the Institution Review Board of the Faculty of Medicine, Chulalongkorn University IRB 009/62, reviewed by the Thai Clinical Trial Registry Committee and prospectively approved for registration 2019-08-08 and Thai Clinical Trial Registry identification number TCTR20190808004. All enrolled participants were provided with information on the study. Written informed consent was obtained from all participants before the start of the study.

### Inclusion and exclusion criteria

All premenopausal and perimenopausal women aged 40–55 years had planned to do hysterectomy with bilateral oophorectomy for benign conditions at King Chulalongkorn Memorial Hospital Thailand were approached. Participant menstrual patterns determined the definition of menopausal status in this study. Premenopausal women in this study were defined by women who had a regular menstrual period in the past 6 months before study enrollment. Women who had menstrual intervals more than 35 days for at least 50% of total cycles in the past year were classified as perimenopause. Exclusion criteria in this study were women with secondary amenorrhea defined by amenorrhea for more than three cycles or 6 months, women with a history of sex hormones or glucocorticoid use within 3 months before study enrollment. Women who had a history or have any conditions known to affect bone turnover markers, including thyroid disorders, parathyroid glands, renal insufficiency, and recent fracture within 12 months were also excluded. Women who had more than one of the particular MHT contraindications such as a history of coronary heart diseases, stroke, venous thrombosis, breast or endometrial cancer, congenital thrombophilia were omitted.

### Sample size justification

The sample size was calculated by the formula of two dependent means. Data from a previous study was used [23, 24]. The α = 0.05 and β = 0.1 were used in the formula. The calculated sample size in this study was 18 participants. When we incorporated a 20% dropout rate, the total sample size required for two dependent tests was 21 participants. We aimed to analyze bone turnover markers in hormone treatment compared with the no-treatment group as the secondary objective. The addition of the same number of participants without hormone treatment was recruited.

### Data collection and intervention

Demographic data was collected. Serum CTX, P1NP, and FSH were obtained at about 7–10 days before elective hysterectomy with oophorectomy (baseline bone turnover markers). Blood was drawn between 8.00 and 9.00 a.m. after an overnight fast for at least 8 h. Serum CTX, P1NP, and FSH were measured by electrochemiluminescence immunoassay (Elecsys kit, Roche Diagnostics Thailand). The inter-assay coefficient of variations (CV) of serum CTX, P1NP, and FSH were 3.8%, 2.3%, and 3.8%, respectively. The intra-assay CV for serum CTX, P1NP, and FSH were 2.1%, 1.8%, and 1.5%, respectively.

Two weeks after operations, the indications for MHT in all participants were assessed by a reproductive endocrinologist. The participants were placed in the treatment or the no-treatment groups according to their symptoms and MHT indications. The primary investigator counseled the participants who had indication(s) for menopausal hormone therapy about the risks and benefits of hormone therapy. The participants who had indication(s) for MHT and were interested in treatment were assigned to the hormone treatment group. They received 2 mg/tab of oral estradiol valerate one tab per day (Progynova ® tablet, the Bayer Thai, Thailand) started at 2 weeks after procedures for a total period of 12 weeks. The participants who had no MHT indication were allocated into the no-treatment group. At the end of 12 weeks, blood was obtained using the same procedure as a baseline. All laboratory measurements were collected on the day after the last pill was taken.

### Outcome measures

The primary outcome was the comparison of serum CTX levels between before and after surgery in each group. The secondary outcomes were comparing CTX and P1NP levels at 12 weeks after surgery between the hormone and no-treatment groups. The other results were to compare serum P1NP levels before and after surgery in each group. Finally, we analyzed the correlation between age at surgical menopause and the baseline serum CTX and P1NP.

### Statistical analysis

IBM SPSS™ statistics version 22.0 for Windows was used for statistical analysis. Descriptive statistics were employed to present baseline data. Comparisons of the median serum CTX and serum P1NP before and after surgical menopause within the group were analyzed by Wilcoxon signed-rank test. Comparisons of the median serum CTX and P1NP at 12 weeks after surgical menopause between the two groups were analyzed by ANCOVA (adjusted for age). Spearman's rank correlation coefficient analysis analyzed the correlation between age and baseline bone turnover markers. Normal distribution of data was tested by Kolmogorov–Smirnov test. A *p*-value of < 0.05 was considered statistically significant.

## Results

### Study participants

**Fig. 1:**
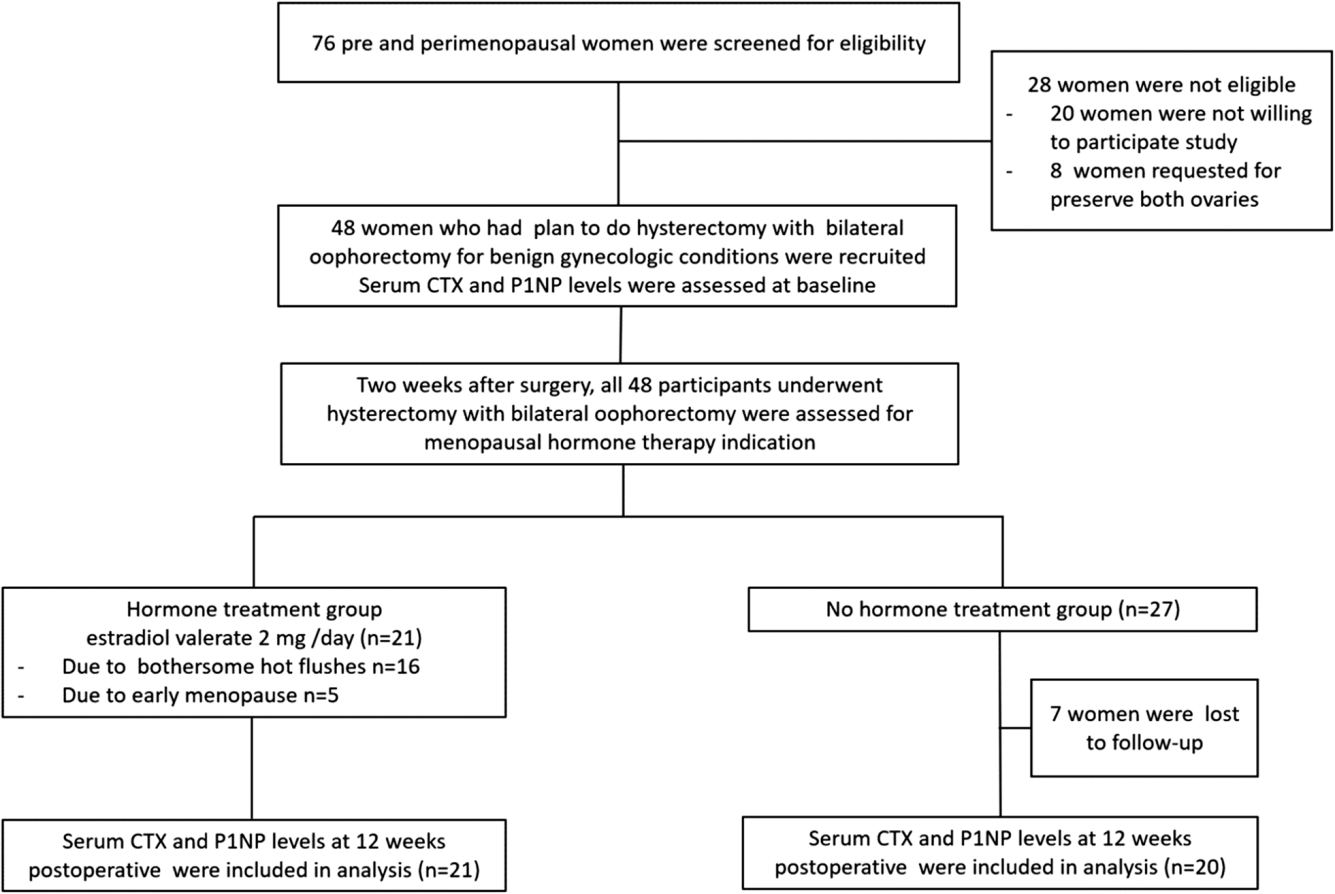
Study flow

From June 2019–January 2020, seventy-six pre and perimenopausal women were assessed for eligibility. Twenty-eight women were not eligible (20 were not willing to participate in the study, and 8 women requested to preserve both ovaries). A total of 48 women met the inclusion criteria and were willing to participate in this study. Only 41 women returned for a follow-up visit at 12 weeks and were included in the analysis. Twenty-one women received 2 mg of oral estradiol valerate per day for 12 weeks. In this hormone treatment group, sixteen women received estradiol valerate due to bothersome vasomotor symptoms, and the others 5 participants received this hormone due to early menopause (premature hypoestrogenic state). There was no participant in this group who denied hormone treatment. All participants in this hormone treatment group continued medication for a total of 12-week period. The others who had no MHT indication were allocated to the no hormone treatment group. There were 27 women in the initial enrollment; seven out of 27 were lost to follow-up. There was no participant in the no treatment group who received any MHT in the study period. Statistical analysis in this study included a total of 41 women who completed a 12-week follow-up period (21 women in the hormone treatment group and 20 women in the no hormone treatment group). The study flow is demonstrated in Fig. 1.

Baseline characteristics of participants are shown in Table 1. There were statistically significant differences in age between the two groups (*p*-value = 0.049). The median (IQR) serum CTX and P1NP levels among women in the hormone treatment group at baseline were 0.21 (0.17–0.35) ng/ml and 37.97 (26.09–54.62) ng/ml, respectively. The median (IQR) serum CTX and P1NP levels in women of no treatment group at baseline were 0.24 (0.19–0.34) ng/ml and 42.11 (35.26–72.57) ng/ml, respectively. There was no statistically significant difference in serum CTX and P1NP levels at baseline between the two groups.

**Table 1:** Baseline characteristics of the participants

	Hormone treatment group (n = 21)	No treatment group (n = 20)	*p*-value
Age* (years)	47.14 (4.1)	49.35 (2.7)	0.049
Age < 45 years	5 (24%)	0 (0%)	
Age ≥ 45 years	16 (76%)	20 (100%)	
BMI* (kg/m^2^)	26.40 (4.9)	23.96 (2.5)	0.056
*Marital status*			
Married	17 (81%)	15 (75%)	
Single	4 (19%)	5 (25%)	
*Parity*			
Nulliparous	9 (43%)	9 (45%)	
Multiparous	12 (57%)	11 (55%)	
*Underlying disease*			
Hypertension	2 (9%)	3 (15%)	
Dyslipidemia	1 (5%)	1 (5%)	
*Primary indication for surgery*			
Myoma uteri	6 (29%)	11 (55%)	
Adenomyosis	4(19%)	6 (30%)	
Endometriosis, endometrioma	10(48%)	4 (20%)	
BRCA mutation	0	1 (5%)	
Endometrial hyperplasia	0	1 (5%)	
CIN III	1 (5%)	0	
FSH** (IU/L)	10.59 (6.84–36.64)	11.86 (4.81–28.30)	0.735
CTX** (ng/ml)	0.21 (0.17–0.35)	0.24 (0.19–0.34)	0.273
P1NP** (ng/ml)	37.97 (26.09–54.62)	42.11 (35.26–72.57)	0.112

^*^Data was presented as mean (SD)

^**^Data was presented as median (IQR)

### Comparison of serum bone turnover markers between baseline and 12 weeks after hysterectomy with bilateral oophorectomy

As the primary objective, a comparison of serum CTX and P1NP levels between baseline and 12 weeks after surgery was evaluated. Among women in the hormone treatment group, median serum CTX and P1NP levels at 12 weeks after surgery were 0.21(0.14–0.34) ng/ml and 42.41(31.42–63.61) ng/ml, respectively. There was no significant difference in CTX and P1NP levels between baseline and 12 weeks after surgery (*p*-value 0.660 and 0.120, respectively). Data are shown in Table 2.

**Table 2:** Comparison of serum bone turnover markers between baseline and 12 weeks after hysterectomy with bilateral oophorectomy

Bone turnover markers	Hormone treatment group (n = 21)			No treatment group (n = 20)		
	At baseline	At 12 weeks	*p*-value	At baseline	At 12 weeks	*p*-value
CTX; ng/ml	0.21 (0.17–0.35)	0.21 (0.14–0.34)	0.660	0.24 (0.19–0.34)	0.47 (0.28–0.65)	< 0.001
P1NP; ng/ml	37.97 (26.09–54.62)	42.41 (31.42–63.61)	0.120	42.11 (35.26–72.57)	63.63 (54.98–80.45)	0.002

Data was presented as median (IQR) and analyzed with Wilcoxon signed rank test

Median serum CTX and P1NP levels among women in the no-treatment group at 12 weeks after surgery were 0.47(0.28–0.65) ng/ml and 63.63 (54.98–80.45) ng/ml, respectively. In contrast with the hormone group, serum CTX and P1NP levels at 12 weeks in no treatment group were significantly higher than baseline (*p*-value < 0.001, 0.002, respectively). Data are shown in Table 2.

### Comparison of serum bone turnovers markers at 12 weeks after hysterectomy with bilateral oophorectomy between the hormone treatment and no treatment groups

As the secondary objectives, we analyzed bone turnover markers 12 weeks after surgery between groups. At 12 weeks after surgery, serum CTX and P1NP levels in hormone treatment group were 0.21 (0.14–0.34) ng/ml and 42.41 (31.42–63.61) ng/ml, respectively. On the contrary, serum CTX and P1NP levels among those in no treatment group were 0.47 (0.28–0.65) ng/ml and 63.63 (54.98–80.45) ng/ml, respectively. Serum CTX and P1NP levels among women in the hormone treatment group were significantly lower than those in the no-treatment group (*p*-value < 0.001, and 0.004), respectively. Data are shown in Table 3. The median serum CTX and P1NP levels at 12 weeks after surgery among women in the hormone treatment group were 55% and 33% of the levels in the no-treatment group, respectively.

**Table 3:** Comparison of serum bone turnovers markers at 12 weeks after hysterectomy with bilateral oophorectomy between the hormone treatment and no treatment groups

Bone turnover markers	Hormone treatment group (n = 21)	No treatment group (n = 20)	*p*-value
CTX levels (ng/ml)	0.21 (0.14–0.34)	0.47 (0.28–0.65)	< 0.001
P1NP levels (ng/ml)	42.41 (31.42–63.61)	63.63 (54.98–80.45)	0.004

Data was presented as median (IQR) and analyzed with ANCOVA (adjusted for age)

### Additional correlation analysis

Due to the possible effects of participant age at the surgery on bone turnover marker levels, we analyzed the correlation between age and bone turnover markers. However, there was no significant correlation between the median serum CTX and age at surgical menopause in both hormone treatment and no treatment group, r = 0.28 *p*-value = 0.22, and r = 0.14 and *p*-value = 0.56, respectively. In the same way, there were no significant correlations between median serum P1NP and age at surgical menopause in both hormone treatment and no treatment group, r = − 0.01 *p*-value = 0.97 and r = 0.08 *p*-value = 0.72, respectively.

### Adverse effects of hormone therapy

There were no significant adverse events of treatment in all participants. Although the relatively high dose of estrogen was prescribed, there was no clinical thromboembolism event, chest pain, syncope, severe gastrointestinal side effects, or drug allergy. However, three women in the hormone treatment group had mild breast pain a few weeks after estrogen initiation. This breast pain symptom was spontaneously improved without medication. These three women continued estrogen therapy until 12 weeks of follow-up.

## Discussion

The present investigation showed a significant elevation of bone turnover markers in surgically menopausal women who did not receive estrogen treatment. Despite a short period, an asymptomatic but significantly high bone resorption process occurred within 3 months after surgery.

Bone remodeling consists of two opposing activities: resorption of old bone by osteoclasts and formation of new bone by osteoblasts. The functions of bone remodeling are replacing old bone, regulating calcium homeostasis, acid–base balance, and releasing growth factors embedded in bone. The bone remodeling process is tightly coupled in time and area at the bone basic multicellular unit (BMU) level [25]. Bone turnover markers release into the bloodstream during the bone remodeling process and provide dynamic information regarding skeletal status.

During bone resorption, collagen is degraded by osteoclasts. CTX, a non-helical fragment of type I collagen-containing cross-linking regions, is released by cathepsin K, an osteoclast-specific protease. The native α‑form of CTX undergoes spontaneous β‑isomerization, which is attributed to protein aging [26]. They circulate in the blood and are partly excreted in the urine. Bone resorption displays circadian variation. CTX shows the highest diurnal amplitude among the BTMs with a peak at 05.00 h and a nadir at 14.00 h. Consumption of breakfast reduces serum CTX by 40% [25]. The secretion of the glucagon-like peptide probably mediates this effect of feeding [27]. Therefore, blood for its measurement must be collected in the fasting status in the morning (between 7 and 10 am). It is inadvisable for some patients (such as those with diabetes) in clinical practice and restricts clinic attendance to morning appointments.

Surgical removal of both ovaries in women before menopause leads to an abrupt declination of circulating estrogen levels [6]. From a previous study, serum CTX was elevated as soon as a month after surgery. Serum CTX levels were continuously elevated until 6 months after surgery. Furthermore, they found a significant negative correlation between bone turnover markers levels and lumbar spine bone mineral density (BMD) at preoperative and 6 months after surgery [14]. In another study, bone resorption and formation markers were raised 3 months after the surgical menopause procedure. However, bone markers levels declined to the baseline levels after menopausal hormone prescription for 3 months [23].

In terms of bone formation marker, we selected serum P1NP as the outcome measurement according to the IOF recommendation. Osteoid, composed of type I collagen, is formed in the early phase of bone formation. Procollagen is a trimer of two α1 and one α2 chain. The PINP is cleaved from procollagen molecule before an assembly of type I collagen molecules into fibers [28]. Although type I collagen is not specific to the bone, most circulating PINP originates from it. P1NP is released into circulation and offers several clinical advantages, including low circadian variation and stability at room temperature. Moreover, P1NP levels are not significantly influenced by dietary intake, and, consequently, patients do not need to be fasting [27]. In a previous study, serum P1NP levels were inversely associated with BMD for the lumbar spine, total hip, and femoral neck even after controlling for age, BMI, and years since menopause. P1NP level was significantly higher in postmenopausal women with osteoporosis compared to those with average bone mass. However, in clinical practice, its low specificity does not warrant utility in osteoporosis diagnosis [29].

Estrogen is essential for the maintenance of sufficient bone mass in reproductive age. Bone resorption and formation were modulated and balanced by circulating estrogen levels. Estrogen activates the synthesis of osteoprotegerin (OPG), the decoy antibodies which neutralize the receptor activator of NF-ĸB ligand (RANKL) and inhibits RANK expression (receptor of RANKL). Responses to estrogen result in inhibition of differentiation and activation of the osteoclasts. Furthermore, estrogen modulates proinflammatory cytokines such as IL-1, IL-6, TNF-α, and PGE2, reducing the pool of osteoclast precursors. The minor estrogenic mechanism on bone is regulated TGF-β expression results in apoptosis of osteoclasts [30]. According to all mechanisms mentioned above, estrogen deprivation is a major detrimental factor on bone physiology. Besides, many studies demonstrated the positive effects of menopausal hormone treatment on bone turnover markers, BMD, and fracture prevention in postmenopausal women [31–34].

As the primary outcome in the present study, there were no notable changes in serum CTX and P1NP levels at 12 weeks in the hormone treatment group compared to baseline. In contrast, serum CTX and P1NP levels were significantly elevated among women who did not receive hormone treatment. In other words, early administration with moderate-dose estrogen could inhibit abnormal bone resorption from acute estrogen deprivation. In secondary outcomes, serum CTX and P1NP levels at 12 weeks after surgical menopause procedure were statistically different between the two groups. The 55% lower median serum CTX level than in the no-treatment group is statistically and clinically significant. The timing of hormone initiation might be an essential issue. In our study, hormone therapy was initiated proximately 2 weeks after surgery. In contrast, Peris et al. study [23] started hormone therapy 3 months post-surgery. The differences between our outcomes and Peris et al.'s finding are partly due to the timing of menopausal hormone initiation.

It should be noted that sixteen out of the total 48 women in our study had moderate to severe hot flushes as early as 2 weeks after oophorectomy. Hence, MHT could be considered as soon as possible in women who has MHT indication. The benefit of MHT in this condition is for improving the quality of life and protecting against accelerated bone loss. However, some clinicians may concern about the risk of venous thrombosis with MHT in the early postoperative period, especially in cases of obesity, metabolic syndrome, and advanced age patients. Transdermal estrogen administration with optimum dose is preferred to minimize the thrombosis risk in these patients.

In terms of treatment effects, we showed that early administration of 2 mg of oral estradiol valerate significantly suppressed the bone remodeling process. However, a conclusion cannot be made for all oral MHT products in the market. Many available products around the world are 1 mg of estradiol plus a variety of progestins. The lower dose of other estradiol products and estrogenic counter-action of various progestins may dramatically affect bone outcomes.

Each participant was evaluated and allocated to the hormone treatment group by FDA-approved MHT indication in the present study. Estradiol valerate 2 mg/day was prescribed for 16 women who had moderate to severe hot flushes and five women diagnosed with early menopause at the time of surgery (age < 45 years). Although early menopause was associated with bone loss in general perception, there was no significant difference in bone turnover markers concentration across quartiles of patient age [29]. To the best of our knowledge, no study confirms a direct association between hot flush symptoms and bone turnover marker concentration. We attenuate selection bias risk by strictly allocating each participant to the hormone treatment group, depending on MHT indication. All participants and a physician assigned treatment did not know baseline bone turnover marker levels at the day of allocation.

Although elevation of bone turnover markers was associated with low BMD and increased risk of fractures, there are many limitations in interpreting bone turnover markers in clinical practices. The biologically interobserver variation, intra-individual variability, analytic reliability, and poorly defined abnormal cut point levels are issues of concern in clinical utility. Vitamin D levels, sunlight exposure, history of fractures over the preceding 12 months, vigorous physical activity, and year should be considered and carefully considered for the interpretation of results. Moreover, the changes in the bone turnover markers are the only representative of bone metabolism; they cannot be used for diagnosing osteoporosis. Dual-energy X-ray absorptiometry for bone density measurement is the standard method used in clinical practice and osteoporosis research. Nowadays, bone turnover markers are primarily used for patients with poor responders, nonadherence to therapy patient identification [35], and can be used as indicators to restart treatment after the bisphosphonate drug holiday period [36].

There are incongruences in data interpretation and recommendations of estrogen therapy and bone, especially for postmenopausal osteoporosis. In the age group 50–60 years or within 10 years after menopause (the window of opportunity concept), the benefits of MHT are most likely to outweigh any risk. Based on the International Menopause Society (IMS) recommendations on women's midlife health and menopause hormone therapy, MHT can be considered first-line therapy in postmenopausal osteoporosis [37]. On the contrary, the North American papers, the American Association of Clinical Endocrinologists/American College of Endocrinology Clinical Practice Guidelines (AACE/ ACE) for Diagnosis and Treatment of Postmenopausal Osteoporosis 2020 stated that estrogen was never approved explicitly for postmenopausal osteoporosis. Estrogen is only approved by the US FDA to prevent postmenopausal osteoporosis and should only be used for women at significant risk of osteoporosis and for whom non-estrogen medications are not considered appropriate [38].

Traditionally routine salpingo-oophorectomy at the time of hysterectomy should be revisited, especially in pre and perimenopausal women, because the lifetime risk of developing ovarian cancer in the general population is only 1 in 70 or 1.4% [39], physicians should make sure that their counseling about risks and benefits is based on current evidence. The reduction of ovarian cancer risk, avoid possible morbidities and future surgery of ovarian disease are the significant potential benefits of salpingo-oophorectomy at the time of hysterectomy. However, these potential benefits must be balanced with the consequences of premature loss of circulating estrogen including, bone loss, hot flushes, cognitive impairment, sexual desire loss, and long-term survival rate [39]. This research emphasized this concept. Forty-nine percent (20/41) of women in our cohort did not receive MHT for bothersome vasomotor symptoms and early menopause indication. These women lost their bone significantly as early as 3 months after surgery. Careful clinical evaluation, lifestyle modification for bone health, and long-term follow-up for bone density and/or quality measurement should be considered. In the present study, we gave patients as much information as possible about the risks and benefits of salpingo-oophorectomy at the time of hysterectomy. Based primarily on patient autonomy, the decisions to do salpingo-oophorectomy were made by participants with adequate information from physicians. In our experiences as a medical school center in Thailand, we found that 30–40% of advanced age premenopausal and perimenopausal women accepted and decided to remove their ovaries at the time of hysterectomy for benign gynecological conditions. However, bone measurement was offered only in a minority of these patients.

Finally, due to the possible effects of participant age on baseline bone turnover marker levels, we made an additional analysis of the correlation between age and bone turnover markers. However, there was no significant correlation between the serum CTX and age at surgical menopause in both hormone treatment and no treatment group, r = 0.28 *p*-value = 0.22, and r = 0.14 and *p*-value = 0.56, respectively. In the same way, there were no significant correlations between serum P1NP and age at surgical menopause in both hormone treatment and no treatment group, r = − 0.01 *p*-value = 0.97 and r = 0.08 *p*-value = 0.72, respectively.

There were limitations of this study. As a nonrandomized design, we could not match the baseline prognostic factors between the two groups. This study type cannot eliminate selection bias. The randomized controlled trial to prove this hypothesis should be considered in further study. Because bone turnover markers can be involved by various factors, such as vitamin D status, sunlight exposure, vigorous physical activity, patient's medical data, and history of recent fractures should be recorded and carefully considered.

## Conclusion

Early estrogen administration with oral estradiol valerate could significantly suppress the high bone remodeling process in surgically induced menopausal women. Further well-controlled studies are required to prove and assess other aspects of bone status with a longer-term follow-up period.

## Data Availability

The datasets used and/or analysed during the current study available from the corresponding author on reasonable request.
